# Investigating the Intentional Google Effect: A Pilot Study Using Antisaccade Eye-Tracking Paradigm

**DOI:** 10.1192/j.eurpsy.2025.936

**Published:** 2025-08-26

**Authors:** A. M. Bukinich, D. A. Bazhenova, G. D. Vzorin, A. M. Konovalova

**Affiliations:** 1 Faculty of Psychology, Lomonosov Moscow State University; 2 Department of Pedagogy and Medical Psychology, Sechenov University, Moscow, Russian Federation

## Abstract

**Introduction:**

There is growing concern that the internet may have a detrimental effect on users, leading to addiction and cognitive decline. One well-known empirical study, conducted by Sparrow, Liu, and Wegner (2011), presented two types of the so-called Google effect (intentional and mnemonic) as evidence. In this study, participants answered either difficult or easy yes/no trivia questions, followed by a Stroop task involving internet-related and neutral words displayed in different colors. The reaction times were slower for internet-related words, but only after the difficult trivia questions, which was interpreted as evidence of automatic internet priming in challenging situations. However, subsequent replication attempts were unsuccessful. We hypothesized that the Stroop paradigm may not be valid due to the lack of semantic interference and that the antisaccade task would be more sensitive.

**Objectives:**

To investigate the intentional Google effect using the antisaccade eye-tracking paradigm.

**Methods:**

A pilot study was conducted (N=37). Participants answered a series of difficult or easy yes/no trivia questions, followed by the appearance of either an internet-related or neutral image on the left or right side of the screen. The task required participants to look in the opposite direction of the stimulus, with oculomotor activity recorded by the SMI Hi-Speed system (1250 Hz). Reaction time and the number of errors were measured.

**Results:**

Logistic regression and ANOVA results did not show a significant influence of question difficulty, stimulus type, or their interaction on error probability (B = -0.22, SE = 0.22, z = -0.94, p = 0.35) or reaction time (F = 0.31, p = 0.58).

**Conclusions:**

These preliminary results do not support the hypothesis for the intentional Google effect, further research and discussion in the context of internet-addiction are needed.

**Disclosure of Interest:**

None Declared

